# Cortical Morphology and White Matter Tractography of Three Phylogenetically Distant Primates: Evidence for a Simian Elaboration

**DOI:** 10.1093/cercor/bhab285

**Published:** 2021-09-13

**Authors:** Lea Roumazeilles, Frederik J Lange, R Austin Benn, Jesper L R Andersson, Mads F Bertelsen, Paul R Manger, Edmund Flach, Alexandre A Khrapitchev, Katherine L Bryant, Jérôme Sallet, Rogier B Mars

**Affiliations:** Wellcome Centre for Integrative Neuroimaging, Department of Experimental Psychology, University of Oxford, Oxford OX13TA, UK; Wellcome Centre for Integrative Neuroimaging, Oxford Centre for Functional MRI of the Brain (FMRIB), Nuffield Department of Clinical Neurosciences, John Radcliffe Hospital, University of Oxford, Oxford OX39DU, UK; Centro Nacional de Investigaciones Cardiovasculares (CNIC), Madrid 28029, Spain; Wellcome Centre for Integrative Neuroimaging, Oxford Centre for Functional MRI of the Brain (FMRIB), Nuffield Department of Clinical Neurosciences, John Radcliffe Hospital, University of Oxford, Oxford OX39DU, UK; Centre for Zoo and Wild Animal Health, Copenhagen Zoo, Frederiksberg 2000, Denmark; School of Anatomical Sciences, Faculty of Health Sciences, University of the Witwatersrand, Johannesburg 2193, South Africa; Wildlife Health Services, Zoological Society of London, London NW14RY, UK (now retired); MRC Oxford Institute for Radiation Oncology, Department of Oncology, University of Oxford, Oxford OX37DQ, UK; Wellcome Centre for Integrative Neuroimaging, Oxford Centre for Functional MRI of the Brain (FMRIB), Nuffield Department of Clinical Neurosciences, John Radcliffe Hospital, University of Oxford, Oxford OX39DU, UK; Wellcome Centre for Integrative Neuroimaging, Department of Experimental Psychology, University of Oxford, Oxford OX13TA, UK; Université Lyon 1, Inserm, Stem Cell and Brain Research Institute U1208, Bron 69500, France; Wellcome Centre for Integrative Neuroimaging, Oxford Centre for Functional MRI of the Brain (FMRIB), Nuffield Department of Clinical Neurosciences, John Radcliffe Hospital, University of Oxford, Oxford OX39DU, UK; Donders Institute for Brain, Cognition and Behaviour, Radboud University Nijmegen, Nijmegen 6525 HR, The Netherlands

**Keywords:** association cortex, association tracts, cercopithecid, connectivity, platyrrhine, strepsirrhine

## Abstract

Comparative neuroimaging has been used to identify changes in white matter architecture across primate species phylogenetically close to humans, but few have compared the phylogenetically distant species. Here, we acquired *postmortem* diffusion imaging data from ring-tailed lemurs (*Lemur catta*), black-capped squirrel monkeys (*Saimiri boliviensis*), and rhesus macaques (*Macaca mulatta*). We were able to establish templates and surfaces allowing us to investigate sulcal, cortical, and white matter anatomy. The results demonstrate an expansion of the frontal projections of the superior longitudinal fasciculus complex in squirrel monkeys and rhesus macaques compared to ring-tailed lemurs, which correlates with sulcal anatomy and the lemur’s smaller prefrontal granular cortex. The connectivity of the ventral pathway in the parietal region is also comparatively reduced in ring-tailed lemurs, with the posterior projections of the inferior longitudinal fasciculus not extending toward parietal cortical areas as in the other species. In the squirrel monkeys we note a very specific occipito-parietal anatomy that is apparent in their surface anatomy and the expansion of the posterior projections of the optical radiation. Our study supports the hypothesis that the connectivity of the prefrontal-parietal regions became relatively elaborated in the simian lineage after divergence from the prosimian lineage.

## Introduction

Comparative neuroscience is an important approach for understanding general brain anatomy and function. Macaques are one of the most commonly studied nonhuman primate species for both ethical and practical reasons (Manger et al. 2008; Perretta 2009). Cercopithecids (Old World monkeys), such as macaques, shared a common ancestor with the Hominoids (the apes including humans) around 25 million years ago (Perelman et al. 2011). With the Platyrrhines (New World monkeys), the Cercopithecids and Hominoids form the infraorder Simiiformes (simian primates), thought to share extensive neuroanatomical similarities. Such similarities are the basis of the translational paradigm in neuroscience, using model species to understand the human brain. However, primates also demonstrate specific mosaic evolution even when correcting for the size of the brain (Smaers and Soligo 2013). Therefore, it is important to compare brain organization in a range of different species to understand how closely the neuroanatomy of traditional animal models parallels human brain organization, but also to elucidate broader principles of neuroanatomical diversity across primates, and to reveal potential species-specific specializations.

Neuroimaging techniques, such as magnetic resonance imaging (MRI), have recently come of age as a tool for whole-brain comparative anatomy (Mars et al. 2014; Rilling 2014). Although MRI provides an indirect quantification of anatomy and is of coarser resolution than classical anatomical techniques, it allows fast, whole-brain quantification of potentially multiple modalities from a single brain (Lerch et al. 2017). These data can, in turn, be related to histological results where they are available (Large et al. 2016; Reveley et al. 2017). Several software packages also permit the reconstruction of cortical brain surfaces from MRI data, which allows investigation of cortical morphology. MRI-based investigations of white matter anatomy in species across the primate order is also facilitated by standardized tools, leading to the creation of white matter atlases of the human, chimpanzee, and macaque brain (Bryant et al. 2020; Warrington et al. 2020).

Comparative neuroimaging studies of white matter architecture in primates have revealed evidence for an expansion of the frontal association tracts in humans compared to other species (Rilling et al. 2008; Barrett et al. 2020; Eichert et al. 2020), as well as more elaborated white matter organization within the great ape ventral visual stream compared to macaques (Roumazeilles et al. 2020); however, such studies have rarely included Platyrrhines and Strepsirrhines (prosimian primates), focusing instead on the Cercopithecids. This is despite the fact that important brain characteristics emerged in Simiiformes, and continued to evolve in this lineage. Such characteristics include additional granular prefrontal cortical areas and more extensive fronto-parietal connectivity (Preuss and Goldman-Rakic 1991; Krubitzer 2009). These novel characteristics have been interpreted as adaptations to specific lifestyles and environments (Passingham and Wise 2012; Genovesio et al. 2014).

Here, we study the neuroanatomy of ring-tailed lemurs *(Lemur catta)*, black-capped squirrel monkeys *(Saimiri boliviensis)*, and rhesus macaques *(Macaca mulatta)*, using high-resolution diffusion MRI in *postmortem* samples. All three species are diurnal primates, live in large multi-male/multi-female groups, and are at least partly arboreal. By taking advantage of newly developed tools, we established robust MRI templates and reconstructed the cortical surfaces. Using tractography in conjunction with the templates we reconstructed white matter tracts for the three species while the surfaces allowed us to visualize their cortical, sulcal, and white matter organization. This enabled us to investigate how changes in brain organization, previously seen across simians, compare to differences with species belonging to other phylogenetic lineages. Specifically, we hypothesized that long-range association tracts might be less extensive in prosimian primates compared to simians.

## Materials and Methods

### Data

For this study we used three *postmortem* brains from each of the following species: ring-tailed lemurs (*L. catta,* between 3 and 11 years, 3 males), black-capped squirrel monkeys (*S. boliviensis,* between 2 and 19 years old, 1 female, 2 males), and rhesus macaques (*M. mulatta,* between 11 and 15 years old, 1 female, 2 males). The samples were obtained from Copenhagen Zoo (lemurs and squirrel monkeys), the Zoological Society of London (squirrel monkey), and the University of Oxford’s Biomedical Sciences (macaques). All brains were extracted and fixed within 24 h after the death of the animal. The brains from the Copenhagen Zoo were obtained after the animals had been euthanized with sodium pentobarbital (intravenous) in line with population management decisions, independent of the current study (Bertelsen 2019). Once euthanized, the carotid arteries were immediately cannulated, and the heads were perfused with an initial rinse of 0.9% saline (1 l/kg) solution at a temperature of 4 °C followed by 4% paraformaldehyde in 0.1 M phosphate buffer (PB) (1 l/kg) at 4 °C. The brains, which showed no signs of neuropathology, were removed from the skull and post-fixed in 4% paraformaldehyde in 0.1 M PB (24 h at 4 °C). The brains were subsequently formalin-fixed in a phosphate-buffered saline (PBS) solution and shipped to Oxford in PBS. The brain from the Zoological Society of London was obtained after the death of the animal from a range of age-related conditions. No evidence of brain pathology was noticed during the necropsy. The brain was fixed in a 10% neutral buffered formalin solution and transported to Oxford in formalin. The brains from the University of Oxford’s Biomedical Sciences were obtained after the animals had been euthanized for reasons unrelated to this study. ﻿Immediately after death, the brains were perfusion fixed with formalin and stored in a 10% neutral buffered formalin solution with azide.

### Imaging Protocol

**Figure 1:**
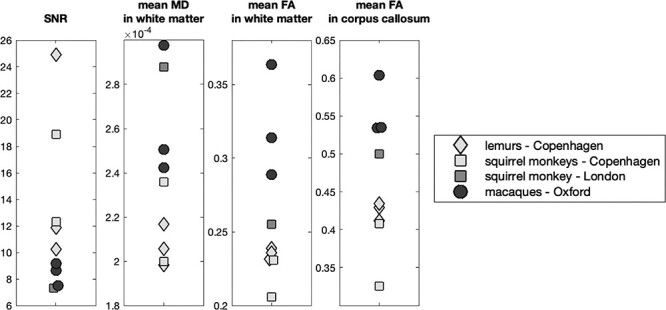
**Data quality assessment.** For each individual, the values of SNR, mean MD, and mean FA in white matter as well as mean FA in the corpus callosum are reported. The marker shape code identifies the different species, while the color code identifies the provenance of the brain as indicated in the legend. FA: fractional anisotropy; MD: mean diffusivity; SNR: signal-to-noise ratio.

All brains were rehydrated in a PBS solution 1 week prior to scanning and placed in fomblin or fluorinert for the scanning procedure. The diffusion-weighted MRI data were acquired from the whole brain using a 7 T preclinical MRI scanner (Varian, Oxford UK). The scanner bore diameter is 210 mm, the gradient coil references are the following: 205_120_HD (Varian, Oxford UK) with a Gmax of 50 G/cm. The radiofrequency coil was made by Rapid Biomedical GmbH (Rimpar, Germany) and is a birdcage transmit receive coil with 72 mm ID. We used a 2D diffusion-weighted spin-echo multi-slice protocol with single line readout (DW-SEMS; TR = 10 s; TE = 26 ms; Matrix size = 128 × 128 with a sufficient number of slices to cover each brain; resolution for lemurs and squirrel monkeys: 0.5 × 0.5 × 0.5 mm^3^, and resolution for macaques: 0.6 × 0.6 × 0.6 mm^3^). A total of 16 non-diffusion-weighted (b = 0 s/mm^2^) and 128 diffusion-weighted (b = 4000 s/mm^2^) volumes were acquired with diffusion encoding directions evenly distributed over the whole sphere (single shell protocol). To assess the data quality, we computed the signal-to-noise ratio (SNR) for each individual scans from the diffusion-weighted volumes. The SNR was defined as the ratio between the mean signal in the brain and the mean signal outside the brain. We also computed the mean fractional anisotropy (FA) and mean diffusivity (MD) in the white matter of each individual, as well as in the corpus callosum. Values for these measures are comparable between individuals, species, and brain providers (Fig. 1).

### Diffusion MRI Data Preprocessing

All data were preprocessed using the same protocol implemented in the module *phoenix* of the MR Comparative Anatomy Toolbox (Mr Cat; www.neuroecologylab.org). Briefly, the steps are as follows: We first converted the datasets to NIFTI format, then built an image based on the volumes acquired without a diffusion gradient as well as a binary mask of this image. Using tools from FSL (www.fmrib.ox.ac.uk/fsl), we then fitted a diffusion tensor model using FSL’s *dtifit* including saving the tensor image which is used in the subsequent template creation. The principal direction image helped to reorient the data to approximate AC/PC (anterior commissure/posterior commissure) conventional orientation which sets the origin at the middle of the anterior commissure, where a small bundle of fibers decussate. Following the preprocessing, *BedpostX* (Behrens et al. 2007) was used to fit a crossing fiber-model to the data, allowing for three fiber orientations.

### Template Creation

We created templates for each of the species using the method described in Lange et al. (2020b). This method employs a registration-based, multi-resolution, iterative template creation strategy including spatial unbiasing of both affine and nonlinear shape changes. Registration is performed using the MultiMOdal Registration Framework (MMORF) (Lange et al. 2020a). MMORF is a multimodal registration tool ﻿for simultaneous alignment of datasets with both scalar and tensor MRI images. Multimodal registration offers advantages over traditional registration algorithms, as it is able to exploit the fact that different imaging modalities provide distinct types of information (e.g., intensity and orientation) and often contain most information at different locations in the brain. Here, we utilized the no-diffusion images with T2 contrast (*nodif*) and the tensor images from FSL’s *dtifit* (*dti_tensor*). As these images were generated from the same raw diffusion-weighted data, they were already co-registered for each individual and have been oriented to approximate the AC/PC convention.

Any residual non-brain tissue remaining after dissection was excluded from the images using a manually defined brain mask in order to avoid artifacts in the resulting templates. The scalar images were intensity bias-field corrected using FSL’s *FAST* tool (Zhang et al. 2001) and globally intensity normalized. Next, a random individual was chosen as an initial template space and all three individual scalar images were then affine registered to this space using *FLIRT* (Jenkinson and Smith 2001). The mid-transformation matrix was obtained to unbias the template toward any individual and the scalar images were then resampled to this unbiased reference and voxelwise averaged across subjects. This averaged image required rigid reorientation to match the AC/PC orientation. The rigid reorienting matrix was combined with the previous affine matrix to obtain our final affine transformation matrix. This was then applied to both scalar and tensor images (using FSL’s *applywarp* and *vecreg* commands, respectively) and the resulting images were averaged across each modalities separately, using a log-tensor averaging for the tensor images, creating our initial scalar and tensor templates. Both images from each subject were then smoothed and simultaneously nonlinearly registered to the initial template at a coarse warp resolution (16 mm isotropic). This process was repeated, doubling the warp resolution every other iteration and reducing the amount of smoothing, for a total of 10 iterations (final warp resolution of 0.5 mm isotropic for lemurs and squirrel monkeys and 0.6 mm isotropic for macaques). At each iteration, the warps describing the transformation from the template space to subject space were spatially unbiased and the resampled images obtained with these warps were averaged as before to create the next template iteration. ﻿MMORF uses a cubic B-spline elastic transformation with mean squared error as the scalar cost function, mean squared Frobenius norm as the tensor cost function, and regularization based on the singular values of the local Jacobian field to ensure warps remain diffeomorphic. The combination of scalar, tensor, and regularization cost functions results in warps which maximize gray and white matter tissue-type overlap, as well as correctly registering location and orientation of white matter bundles, while adhering to biologically plausible set of deformation constraints.

Each individual fractional anisotropy image and mean principal diffusion direction distribution (in vector form, output of *BedpostX*) were transformed to the template space using the *applywarp* and *vercreg* FSL commands, respectively. We then averaged these images across individuals of the same species, to obtain mean principal diffusion image and mean fractional anisotropy image in template space.

### Surface Creation and Labeling

Surfaces were generated with the preclinical surface pipeline *precon_all (*https://github.com/neurabenn/precon_all). *precon_all* is a minimalist adaptation of Freesurfer’s (Fischl 2012) recon-all pipeline, aiming to provide broad flexibility to reconstruct cortical surface meshes without a known segmentation or parcellation scheme. This allows *precon_all* to generate cortical surface meshes in lesser studied animal models. It consists of a modularly designed pipeline and can run brain extraction, tissue segmentation, white matter filling, and surface generation in a continuous workflow on images with a T1-like contrast. To accommodate this, we converted the T2 contrast of our templates to obtain T1-like contrast images, using FSL tools. We inverted the intensities by multiplying the template image by −1 and adding the maximum intensity of the initial image, making sure the cerebrospinal fluid and ventricle intensities remained at zero.

In all three templates, we ran *precon_all* twice; the first run used the automated segmentation from ANTs Atropos (Avants et al. 2009), and the second used a hand-edited WM mask. The WM segmentation was filled with hand-drawn “subcortical” and “non-cortical” masks. The subcortical mask is an inclusion mask and begins at the superior border of the corpus callosum and fills the subcortex between the outer borders of the left and right lateral ventricles. The non-cortical mask lies directly posterior and inferior to the subcortical mask and includes the cerebellum and brainstem. Both subcortical and non-cortical masks were drawn using ITK-SNAP (www.itksnap.org, Yushkevich et al. 2006). The filling process uses these masks to remove the cerebellum and brainstem and fill the subcortex between the lateral ventricles beneath the corpus callosum. This created the prerequisite volumetric image that can be used to create white, pial, and midthickness surfaces. The final surfaces presented here all use a hand-edited WM mask and they were downsampled in connectome workbench to a normal sphere with 10 242 vertices.

For visual comparison with known anatomical regions, we also labeled sulci and specific areas on the cortical surface. Major sulci were labeled on the cortical surface of the rhesus macaque and squirrel monkey using the terminology used for the macaque by Petrides (2005, 2011). For the ring-tailed lemurs, apparent similarities in sulcal patterns could be observed but only few studies existed on this genus with a lack of consensus on the labeling to be used for some sulci (Mott and Kelley 1908; Brodmann 1909; Connolly 1936; Radinsky 1975). Therefore, to be consistent with the macaque terminology, we refer to the ambiguous lemur sulci with a “l” prefix to reflect on their probable homology or at least the shared topography with the macaque sulci.

Cortical labels were drawn from previous illustrations on the surface using Connectome Workbench tools (Marcus et al. 2011). For the lemur, the primary visual (V1) and motor cortex (M1) borders were based on illustrations from histological studies (Mott and Kelley 1908; Fasemore et al. 2018). For the squirrel monkey these borders were based on the ﻿VALiDATe29 atlas, providing surface borders based on histological data (Schilling et al. 2017). For the macaque, V1 and M1 borders were based on several histological atlases that have been adapted to standard macaque surface (von Bonin and Bailey 1947; Paxinos et al. 2000). For the prefrontal (PF) cortex, we used the definition from Passingham and Wise (2012) which defines PF as all the areas anterior to any of the premotor and supplementary motor areas. For PF and granular areas in the macaque and squirrel monkey, we used illustrations from this book (Passingham and Wise 2012), completed by illustrations from another squirrel monkey source (Rosabal 1967). There exists only a limited amount of studies about the ring-tailed lemur prefrontal cortex, therefore we combined illustrations of another prosimian, the bushbaby (Preuss and Goldman-Rakic 1991), with the previously mentioned lemurs illustrations (Mott and Kelley 1908) to estimate the extent of the lemur prefrontal cortex and its granular areas.

### Tractography

The tracts were reconstructed using probabilistic tractography as implemented in the *Xtract* tool (Warrington et al. 2020). We defined tractography seed, target, and exclusion masks in the template space of each species. These were then transformed to the diffusion space of each individual using the warps obtained during the template creation. We defined all masks to be in as similar an anatomical position as possible in all three species, based on anatomical landmarks (see details below). The tractography algorithm starts from the seed, the streamlines follow local orientations sampled from the posterior distribution given by *BedpostX* and only the streamlines that reached or passed through the target and not through the exclusion mask were conserved. All the *Xtract* options were the default, only the step length was adjusted to 0.2 mm to reflect the small voxel and brain size of our data. In each seed voxel 1000 samples were seeded. The output of the tractography is a tractogram image which represents the fiber probability distribution. For all protocols, a second tractography was run inverting the roles of the seed and target and the resulting tractogram of the two protocols were added and normalized ﻿by dividing the path distribution by the total number of generated streamlines not rejected by target or exclusion mask criteria. This normalized tractogram obtained for each subject was transformed back to the template space, averaged across subjects and log-transformed to obtain a value between zero and one, facilitating the threshold determination. We used a threshold of 0.8 which resulted in selecting the most similar higher densities of streamlines reaching each voxel for all tracts and all species.

**Figure 2:**
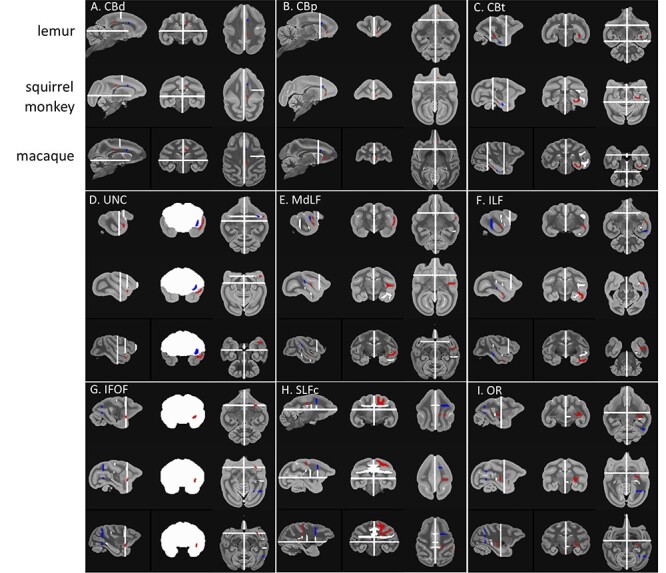
**Tractography recipes for lemurs, squirrel monkeys, and macaques.** The seed mask (red), the target mask (blue), and the exclusion mask (white) are represented for the left hemisphere protocols on the template image for each species in radiological convention.

We will now detail the tractography recipes used for each tract (Fig. 2). The recipes are based on the *Xtract* recipes for the macaque and previous literature (Bryant et al. 2020, 2021). As all the tracts reconstructed here are unilateral, all exclusion masks contain a sagittal plane at the midline to avoid streamlines crossing to the other hemisphere.

*Cingulum bundle (CB):* The CB has previously been segmented based on the presence of fibers connecting specific targets (Heilbronner and Haber 2014). This segmentation has informed previous tractography protocols reconstructing the CB in primates leading to the adaptation of a three sections protocol to capture the entirety of the CB (Bryant et al. 2020; Warrington et al. 2020). We reconstructed the CB in three different sections: dorsal (CBd), peri-genual (CBp), and temporal (CBt). The seed and target of the CBd were placed in the white matter of the cingulum gyrus. Dividing the corpus callosum in three equal segments, the seed of the CBd was placed at the front of the most posterior segment (Fig. 2*A*). Its target was placed at the start of the genu of the corpus callosum. The exclusion mask was made of a coronal mask through the territory of the SLFc at the level of the midpoint of the corpus callosum and an axial mask below the corpus callosum to avoid invading the SLFc territory and the CBt, respectively. The seed of the CBp was placed at the ventral terminal point of the genu of the corpus callosum and its target in the dorsal end of the genu (Fig. 2*B*). The exclusion mask was made of a coronal plane just anterior to the temporal lobe to restrict the tractography to this specific CB section. The seed and target of the CBt were placed in the white matter just inferior to the parahippocampal gyrus posterior and anterior, respectively (Fig. 2*C*). The exclusion mask was made of a coronal plane at the level of the midpoint of the extreme/external capsule, a coronal plane posterior to the corpus callosum and the seeds and targets of the MdLF and ILF to avoid invading occipitotemporal or extreme/external capsule fibers.

*Uncinate fasciculus (UNC):* The UNC was reconstructed by placing a seed in the superior temporal gyrus where the temporal and frontal cortices are first separated and a target in the same coronal section but in the ventral part of the extreme capsule (Fig. 2*D*). The exclusion mask was made of a frontal coronal section at the level of the seed and target but excluding the seed and target, a posterior coronal section to avoid invading the temporal tracts, and a frontal dorsal coronal section to avoid invading dorsal tracts.

*Middle longitudinal fasciculus (MdLF):* The MdLF was reconstructed by placing a seed in the superior temporal gyrus on a coronal slice slightly anterior to the central sulcus (Fig. 2*E*). Its target was placed posteriorly in the superior temporal gyrus, just anterior to the posterior terminus of the Sylvian fissure. The exclusion mask was made of a frontal coronal mask through the extreme capsule and seeds and targets from the ILF, CBt, and IFOF to prevent leakage in these fibers.

*Inferior longitudinal fasciculus (ILF):* The ILF was reconstructed by placing a seed in the anterior inferior temporal gyrus on a coronal slice slightly anterior to the central sulcus (Fig. 2*F*). Its target was placed posteriorly in the inferior temporal gyrus, just anterior to the posterior terminus of the Sylvian fissure. The exclusion mask was made of a frontal coronal mask through the extreme capsule and seeds and targets from the MdLF, CBt, and IFOF to prevent invading these fibers.

*Inferior fronto-occipital fasciculus (IFOF):* We reconstructed the IFOF by placing a seed in the extreme capsule where it connects temporal and frontal cortex and a target as a coronal plane just anterior to the lunate sulcus (Fig. 2*G*). The exclusion mask was made of a coronal mask encompassing the whole white matter except the seed at the level of the seed to avoid spurious anterior–posterior fibers and the seeds and targets from MdLF, ILF, and SLFc to prevent invading these tracts.

*Superior longitudinal fasciculus complex (SLFc):* We reconstructed the SLFc as a complex of the three SLF branches (Thiebaut de Schotten et al. 2011). The seed was defined as a large coronal mask in the parietal cortex immediately posterior to the dorsal end of the central sulcus (Fig. 2*H*). The target was also a large coronal mask in the territory of the SLFc at the level of the anterior commissure. The exclusion mask was made of an axial exclusion below the corpus callosum to avoid invading temporal tracts, and three coronal masks in the cingulate gyrus, two of them at the same coronal level as the seed and target and one in between, in the region of midpoint of the central sulcus, to avoid invading the cingulum bundle. Three additional coronal exclude masks were placed at the same level in the external and internal capsule to avoid invading these fibers. Finally the seeds and targets from the MdLF recipe were added to the exclusion to make sure the SLFc did not invade ventral pathways.

*Optic radiation (OR):* We reconstructed the OR by placing a seed in the lateral geniculate nucleus and a target as a coronal plane just anterior to the lunate sulcus (Fig. 2*I*). The exclusion consisted of an axial block of the brainstem, a coronal block directly posterior to the LGN to select only fibers that curl around dorsally, and a coronal plane just anterior to the seed to prevent invading the longitudinal fibers.

We used a Matlab in-house routine to produce three-dimensional visualization of the averaged log-transformed tractograms for each species, to facilitate the study and the representation of the whole tract anatomy (as seen in subpanel A of tract result figure).

**Figure 3:**
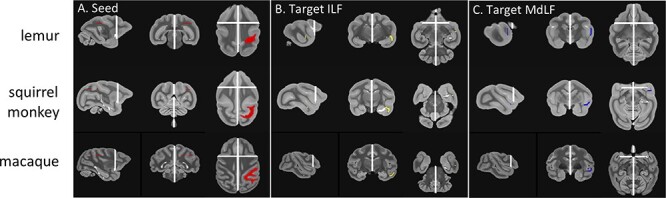
**Additional tractography recipes for lemurs, squirrel monkeys, and macaques.** (*A*) The seed mask (red), (*B*) the target mask for ILF (yellow), (*C*) MdLF (blue), and the exclusion mask (white) are represented for the left hemisphere protocols on the template image for each species in radiological convention.

We ran two additional control analyses. The first aimed to confirm that the ILF did not reach parietal cortex in the lemur. Our previous protocol reconstructed the tracts based on seeds and targets placed in the core of the tract, letting the extremities be defined by the tractography algorithm. For this control, we therefore used a modified protocol in which we placed the seed at the posterior extremity in the parietal cortex and the target at the anterior extremity in the temporal pole and assessed the likelihood of reconstructing a white matter bundle with such protocol. We used the MdLF for comparison as it reaches the parietal cortex in all three species and runs parallel to the ILF in the temporal lobe. The modified protocols used the same seed and exclusion for both ILF and MLF, only the targets changed. In more details, the common large seed was placed in the axial plane encompassing the ventral posterior parietal cortex of the three species (Fig. 3*A*). We used the same exclusion masks as previously defined for the main tractography of MdLF and ILF except that we did not include the seeds and targets of these tracts. The ILF target was placed in the anterior inferior temporal gyrus (Fig. 3*B*) and the MdLF target in the anterior superior temporal gyrus (Fig. 3*C*). These targets contained the same number of voxels for ILF and MdLF in all species. To account for the plausibility of the tracts, we then computed the ratio of streamlines surviving these two tractography protocols. If only a few streamlines survive the target and exclusion criteria, it means that a tractography protocol is unlikely to reconstruct one of the major white matter bundles, such as ILF and MdLF. Therefore, by comparing the number of surviving streamlines with these two similar protocols we can assess how likely the ILF reaches the parietal cortex. If the ratio of streamlines surviving with the MdLF target compared to the ILF is very high, it means that MdLF reaches parietal cortex more than ILF and vice versa.

The second control analysis aimed to confirm the inter-species findings concerning the SLFc and OR. To ensure these effects are not due to changes in overall brain size, we aimed to show that tracts running in similar parts of the brain showed dissociable changes between species. If this proved to be the case, our results cannot simply be ascribed to differences due to overall scaling of the white matter. From the results of the initial tractography, we have noticed that the CB, an evolutionarily conserved tract (Bubb et al. 2018) follows a similar course across species; its dorsal component runs through the dorsal part of the cortex parallel to the SLFc, while its temporal component runs close to part of OR. Therefore, we calculated the ratio between the number of voxels reached by SLFc and OR and the number of voxels reached by the CB segment running in a similar part of the cortex.

### Surface Projection Maps

Cortical surface representations were obtained for each tract of each species to investigate the cortical territory reached by the tracts. We used a recently developed approach to reduce the issues caused by gyral bias and superficial white matter (Reveley et al. 2015). This approach is to multiply the tractograms obtained with a whole brain connectivity matrix (Mars et al. 2018). For each species, we obtained an average matrix across subjects representing the connectivity between all the vertices of the cortical surface and all the voxels in the brain volume. We used the brain extracted template image and the template surface generated above for each species. Each of the tracts from the tractography (not log normalized or thresholded) was multiplied to this average matrix, to obtain a map representing their connectivity with the cortical surface. The result was smoothed using a Gaussian surface smoothing kernel of 1 mm using the *cifti-smoothing* command from Connectome Workbench (Marcus et al. 2011) and log transformed and thresholded at 0.8 as per the tractography result.

### Data Availability Statement

Raw data of the lemur and squirrel monkey will be made available on the Digital Brain Bank of the Wellcome Centre for Integrative Neuroimaging (https://open.win.ox.ac.uk/DigitalBrainBank/#/) upon acceptance of the paper. Macaque data are already available within PRIME-DE (Milham et al. 2018); http://fcon_1000.projects.nitrc.org/indi/indiPRIME.html). The scripts used for preprocessing are available in MrCat (www.neuroecologylab.org, https://github.com/neuroecology/MrCat). MMORF is available as a Singularity image online (https://git.fmrib.ox.ac.uk/flange/mmorf_beta). The surface pipeline is available online (https://github.com/neurabenn/precon_all). Template and surface images, tractography recipes and results, template and analysis code are available online (https://git.fmrib.ox.ac.uk/rlea/small_primate_brains).

## Results

In this study, we acquired diffusion MRI data from three primate species representing three distinct lineages: strepsirrhines (ring-tailed lemur), platyrrhines (black-capped squirrel monkey), and cercopithecids (rhesus macaque). The dataset is of high quality and three individual brains were used for each of the species, allowing us to establish templates using a recently presented multimodal registration method (Lange et al. 2020b), and surfaces using a modified version of previous tools (Benn et al. 2020). In turn, these allowed us to investigate and compare the cortical, sulcal, and white matter anatomy of the three species.

### Cortical Surface

**Figure 4:**
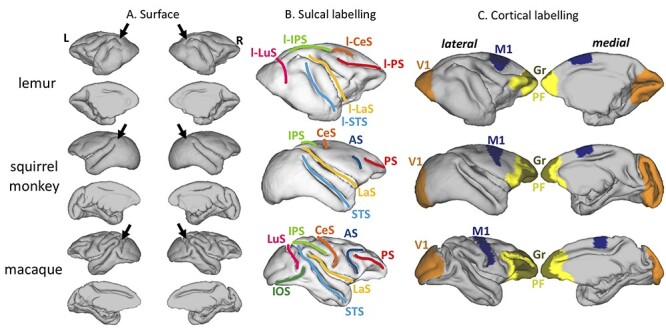
**Cortical surface labeling.** (*A*) Pial cortical surfaces of ring-tailed lemurs, black-capped squirrel monkeys, and rhesus macaques obtained from the template. The arrows indicate the dorsal most location of the occipito-parietal cortex in the three species. (*B*) Labeling of the sulci on the lateral mid-thickness surfaces. AS: arcuate sulcus; CeS: central sulcus; IOS: inferior occipital sulcus; IPS: intra-parietal sulcus; LaS: lateral sulcus; LuS: lunate sulcus; PS: principal sulcus; STS: superior temporal sulcus. (*C*) Labeling of the primary visual cortex (V1), primary motor cortex (M1), prefrontal cortex (PF) and its granular areas (Gr) on the right hemisphere mid-thickness cortical surfaces.

Cortical surface reconstructions reveal that all three species show some degree of cortical folding (Fig. 4*A*), but this is most apparent in the macaque, with the squirrel monkey showing the least evidence for deep sulci in the occipital, and to a lesser extent, frontal cortex. Due to the lack of consensus in the literature, we adapted the macaque terminology and added a “l” prefix for the ring-tailed lemur sulci. The squirrel monkey cortical surface shows a very lissencephalic anatomy with only two very pronounced sulci, the lateral sulcus (LaS, also called Sylvian fissure), and the superior temporal sulcus (STS) (Fig. 4*B*). We could also observe three other sulci in all three species: the principal sulcus (PS), the central sulcus (CeS), and the intra-parietal sulcus (IPS). However, we are cautious in labeling these sulci in lemurs as previous reports have disagreed in their labeling. It is evident from the surface reconstruction that the PS extends more posteriorly in lemurs than in the other species, even merging with the IPS. This had been previously interpreted as a unique sulcus called the coronal sulcus, similar to what is observed in non-primate mammals (Radinsky 1975), while others have kept them separated (Mott and Kelley 1908). Others have argued that the lemur CeS is actually formed by both the CeS (as indicated here) and the posterior portion of the PS (Connolly 1936). Posteriorly, the IPS is substantially more extensive in lemurs and macaques than in squirrel monkeys. The border between occipital and parietal cortex (marked with an arrow in Fig. 4*A*) suggests that much more of the dorso-caudal surface of the occipital lobe is occupied by visual cortex in squirrel monkeys than in the other species studied.

To put this cortical labeling in context, it is helpful to compare the sulcal anatomy to known cytoarchitectonic subdivisions in the three species. We labeled primary visual cortex (V1), primary motor cortex (M1), prefrontal cortex (PF), and the granular part of prefrontal cortex (Gr) in the three species (Fig. 4*C*) based on existing anatomical atlases (Mott and Kelley 1908; von Bonin and Bailey 1947; Paxinos et al. 2000; Schilling et al. 2017), and found that the morphology and cortical territory of V1 differs substantially across species. V1 shows a very different folding pattern in lemurs compared to the two other species, apparent in the dorsally rotated orientation of the calcarine sulcus. Furthermore, V1 occupies proportionally more cortical surface area in the squirrel monkey (30% of total surface in squirrel monkeys, against 21% and 15%, respectively, for lemurs and macaques). The expansion of PF in squirrel monkeys and rhesus macaques can be illustrated by comparing on the medial surface the locations and sizes of M1 and PF, which appear further apart and PF covers more areas in squirrel monkeys and rhesus macaques than in ring-tailed lemurs. The large arcuate sulcus in rhesus macaques also increases the cortical territory of PF on the lateral surface. PF contains more granular territory in squirrel monkeys and rhesus macaques compared to that of ring-tailed lemurs, as is confirmed by the ratios of granular surface to prefrontal surface: 15% in lemurs compared to 30% and 43%, respectively, in squirrel monkeys and rhesus macaques.

### White Matter Anatomy

**Figure 5:**
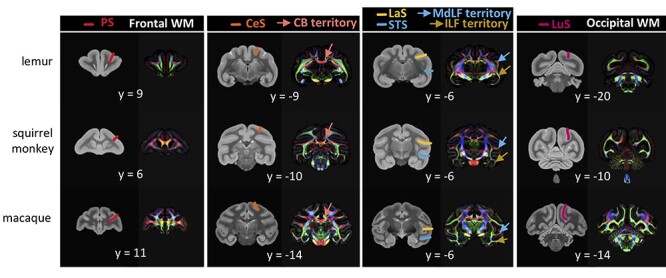
**Anatomical landmarks for white matter tract definition.** Sulcal landmarks on the template image (left) and the principal diffusion directions modulated by the fractional anisotropy (right) are shown for ring-tailed lemurs, squirrel monkeys, and rhesus macaques in radiological convention. The lemur sulci correspond to the sulci with the “l” prefix as in Fig. 4. Colors of the principal diffusion images indicate the directions: red left–right, green anterior–posterior, and blue dorsal-ventral. CB: cingulum bundle; CeS: central sulcus; ILF: inferior longitudinal fasciculus; LaS: lateral sulcus; LuS: lunate sulcus; MdLF: middle longitudinal fasciculus; PS: principal sulcus; STS: superior temporal sulcus; WM: white matter.

We reconstructed several white matter tracts using probabilistic tractography. We employed similar recipes in the three species based on common anatomical features and principal direction images identifying landmarks for white matter tract definition (Fig. 5). All the tracts are displayed as a 3D reconstruction and as a projection to the cortical surface.

**Figure 6:**
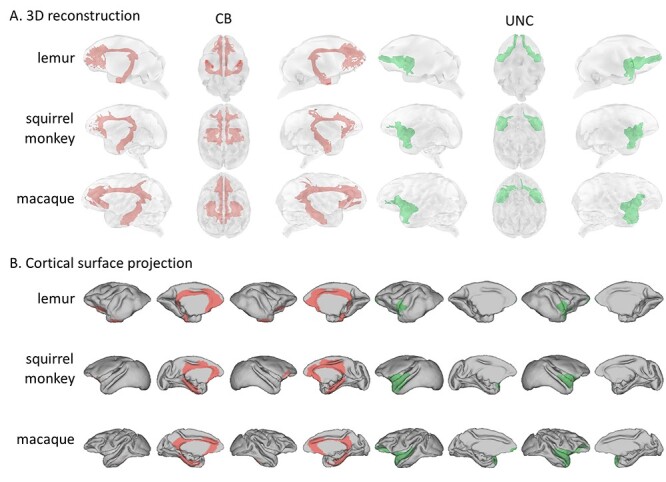
**Tractography results for the CB and UNC.** (*A*) We represented the averaged log transformed, thresholded tractogram with a 3D reconstruction showing from left to right: left hemisphere, ventral view, right hemisphere. (*B*) The projections to the mid-thickness cortical surface show left and right hemispheres from both lateral and medial views.

From the limbic tracts, we reconstructed the cingulum bundle (CB). This is a tract extending from the para hippocampal gyrus, through medial posterior temporal lobe, coursing rostro-caudally superior to the corpus callosum and terminating in medial prefrontal cortex. Because of the sharp curvature of this tract, we reconstructed it in three different sections: peri-genual (CBp), dorsal (CBd), and temporal (CBt). We were able to obtain the whole CB with a very similar anatomy showing prefrontal projections in the three species, while also showing some posterior parietal projections in squirrel monkeys and rhesus macaques (Fig. 6). It also appears that the posterior end of the dorsal segment is located relatively more rostral in squirrel monkeys compared to rhesus macaques, possibly because of the difference in the anatomy of the visual cortex described above.

From the temporal lobe tracts, we reconstructed the uncinate fasciculus (UNC), the middle longitudinal fasciculus (MdLF), the inferior longitudinal fasciculus (ILF), and the inferior fronto-occipital fasciculus (IFOF).

The UNC is a tract that connects the temporal pole to the medial and orbital prefrontal cortex *via* the extreme/external capsule. The UNC is also considered a limbic tract (Alves et al. 2019). The anatomy of the uncinate is quite similar in the three species studied. Although it has been shown previously that few fibers reach the frontal pole in macaques as well (Schmahmann et al. 2007), we observe more streamlines reaching the rostral prefrontal cortex in the ring-tailed lemur compared to both the rhesus macaque and squirrel monkey, possibly because of the reduced granular prefrontal cortex in the lemur (Fig. 6). These observations suggest that similar cortical areas are innervated by this tract. The difference observed highlights the expansion of granular prefrontal cortex in the lineage to which squirrel monkeys and rhesus macaques belong and the relative position of prefrontal cortex areas in the three species (Passingham and Wise 2012).

**Figure 7:**
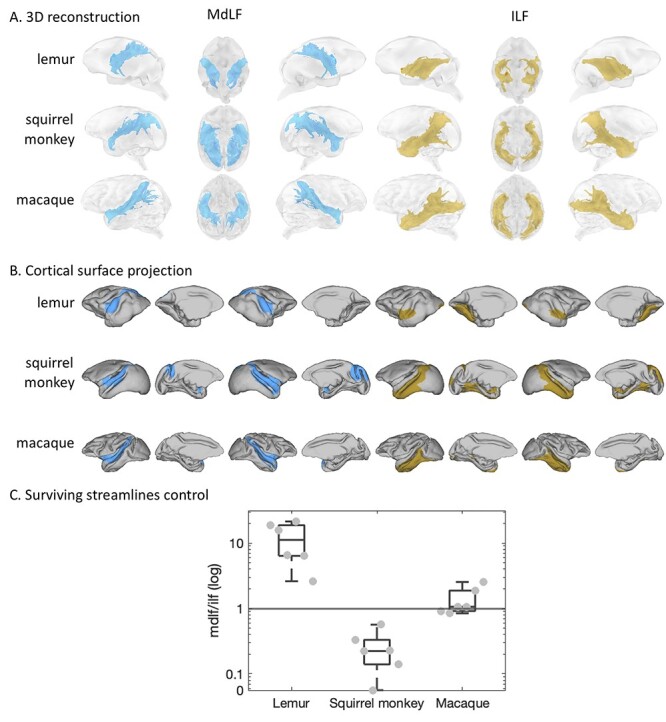
**Tractography results for the MdLF and ILF.** (*A*) We represented the averaged log transformed, thresholded tractogram with a 3D reconstruction showing from left to right: left hemisphere, ventral view, right hemisphere. (*B*) The projections to the mid-thickness cortical surface show left and right hemispheres from both lateral and medial views. (*C*) The boxplot shows the log ratio of surviving streamlines between control protocols using the MdLF or the ILF target. On each box, the central mark indicates the median, and the bottom and top edges of the box indicate the 25th and 75th percentiles, respectively. The whiskers extend to the most extreme data points not considered outliers. Each gray dot represents the ratio for one hemisphere from one individual.

The MdLF is a longitudinal tract spanning the length of the superior temporal gyrus and projecting to the occipital and posterior parietal cortex. It has been shown to exhibit a conserved and similar anatomy in rhesus macaques, great apes such as chimpanzees and humans (Bryant et al. 2020; Roumazeilles et al. 2020). The anatomy of the MdLF is also very similar in ring-tailed lemurs, squirrel monkeys, and rhesus macaques studied herein, with the only difference occurring in the somewhat broader projections to the occipital cortex in squirrel monkeys compared to the other species (Fig. 7*A–B*).

The ILF is a longitudinal tract running parallel to the MdLF and it has been described in macaques as spanning the length of the inferior temporal gyrus from the temporal pole to occipital and posterior parietal regions (Schmahmann et al. 2007). However, our tractography protocol, although similar in all species, could not identify a parietal projection in ring-tailed lemurs even though it was present in both squirrel monkeys and rhesus macaques (Fig. 7*A–B*). To verify this result was not due to issues in the tractography protocols, we performed an additional tractography analysis, using a large seed encompassing the parietal cortex and targets either in the territory of the anterior MdLF or the anterior ILF. We calculated the number of surviving streamlines with these two tractography protocols for each individual and each hemisphere, to investigate the plausibility of a tract running from the parietal cortex to these two anterior temporal locations. We then calculated the ratio between these surviving streamlines for MdLF and ILF protocols. The results confirmed the observation from the initial tractography. Seeding in ring-tailed lemur parietal cortex showed only minimal fibers reaching the ILF running through the inferior temporal gyrus compared to fibers reaching the MdLF running through the superior temporal gyrus (log ratio around 10) (Fig. 7*C*). In contrast, from the parietal cortex of the squirrel monkey, the MdLF was less likely to be reached than the ILF (log ratio close to 0), and the rhesus macaque showed similar connectivity of parietal with MdLF and ILF (log ratio close to 1). This result suggests parietal connectivity of the ILF in squirrel monkeys and rhesus macaques, not present in ring-tailed lemurs.

**Figure 8:**
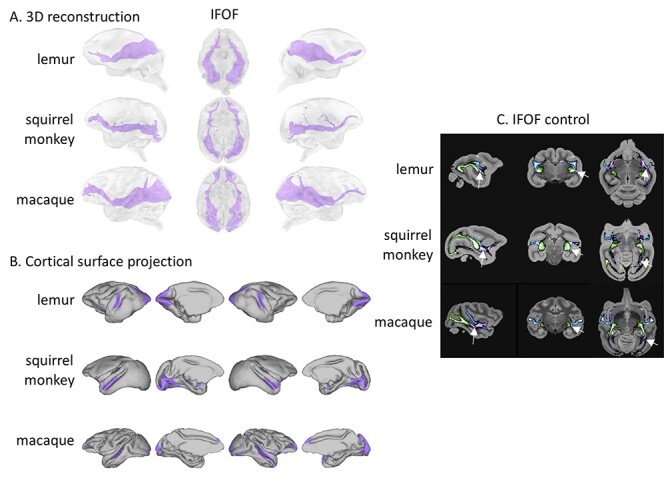
**Tractography results for the IFOF.** (*A*) We represented the averaged log transformed, thresholded tractogram with a 3D reconstruction showing from left to right: left hemisphere, ventral view, right hemisphere. (*B*) The projections to the mid-thickness cortical surface show left and right hemispheres from both lateral and medial views. (*C*) The arrows on the sagittal, coronal, and axial slices point to locations where the IFOF (purple) path is clearly separated from the MdLF (blue) and OR (green). The tracts are shown on the template in radiological convention.

The presence of an inferior fronto-occipital fasciculus (IFOF), also called the longitudinal fronto-temporal tract or extreme capsule complex, has been established in several species of cercopithecids using both tractography and blunt dissection (Mars et al. 2016; Schaeffer et al. 2017; Decramer et al. 2018; Barrett et al. 2020; Bryant et al. 2020; Roumazeilles et al. 2020). This multisynaptic tract has been described as connecting the occipital lobe and the frontal lobe *via* the temporal lobe and the extreme/external capsule. Its overall shape appears very similar in all three species studied herein, although its frontal projections appear more robust toward the PF in squirrel monkeys and rhesus macaques than in ring-tailed lemurs, and its occipital projections appear more restricted in squirrel monkeys, particularly compared with the OR projections in that region of the brain (Fig. 8*A–B*). Sagittal, coronal, and axial brain sections of the three species showing the OR, MdLF, and IFOF reinforce the argument that IFOF is a tract that is distinct from other tracts occupying the same region (Fig. 8*C*).

**Figure 9:**
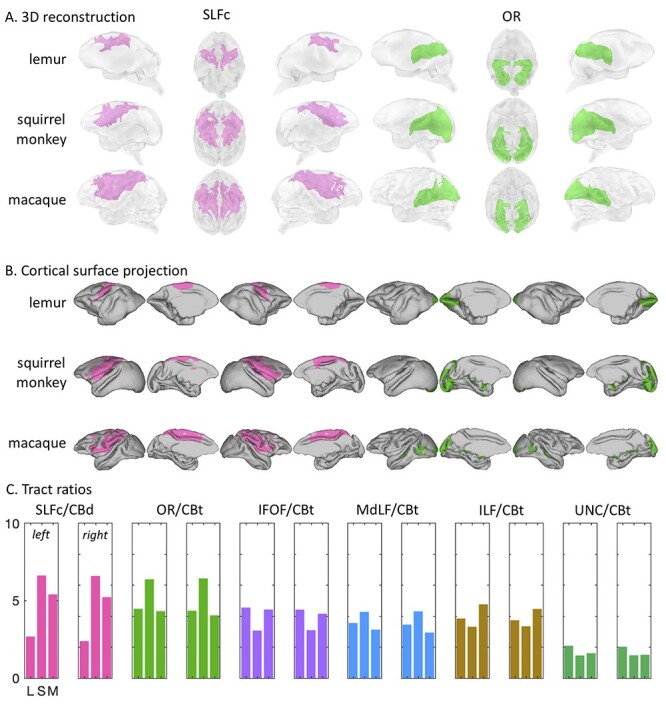
**Tractography results for the SLFc and OR.** (*A*) We represented the averaged log transformed, thresholded tractogram with a 3D reconstruction showing from left to right: left hemisphere, ventral view, right hemisphere. (*B*) The projections to the mid-thickness cortical surface show left and right hemispheres from both lateral and medial views. (*C*) Tract ratios with the CBd and CBt for the tracts of interest: SLFc and OR, as well as the rest of the tracts. Both left and right hemisphere ratios are represented. L: lemurs; S: squirrel monkeys; M: macaques.

**Figure 10:**
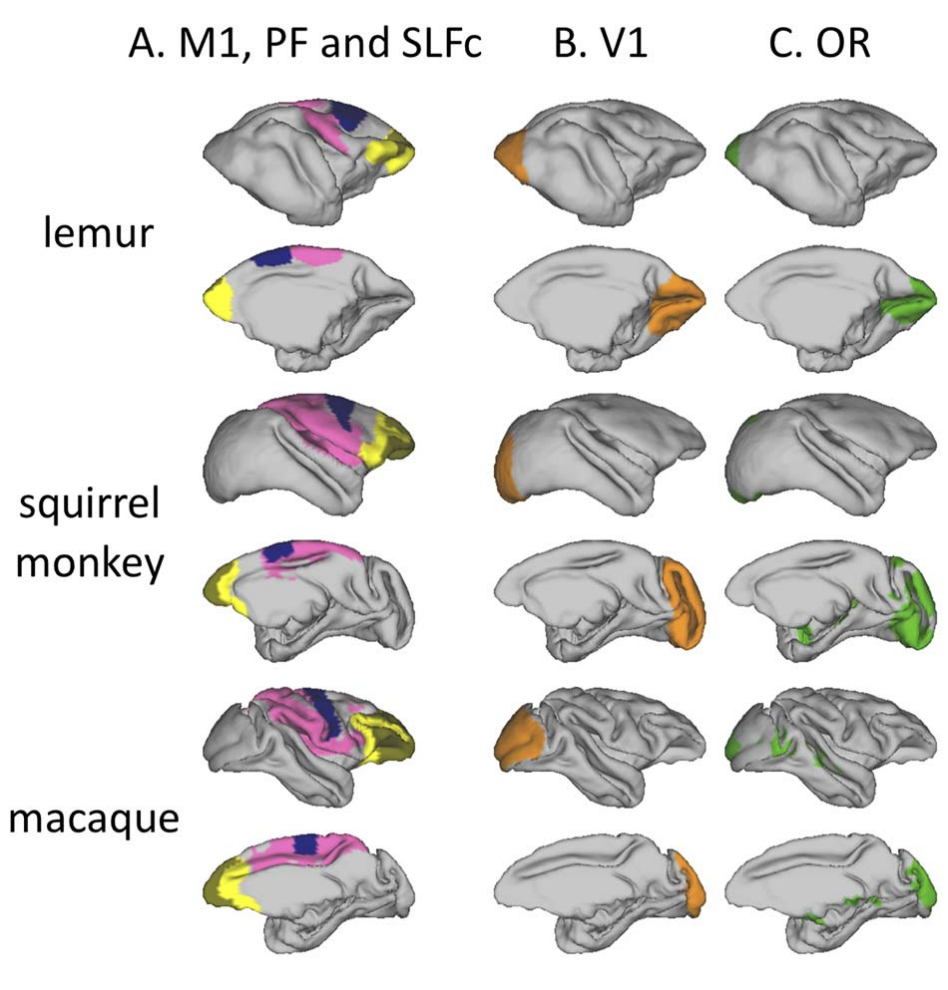
**Cortical surface labeling and tractography.** (*A*) Right hemisphere mid-thickness cortical surface showing M1 (blue), PF and granular areas (light and dark yellow, respectively), and SLFc projections (pink). (*B*) Right hemisphere mid-thickness cortical surface showing V1 (orange) and (*C*) showing OR projections (green).

In the dorsal portion of the telencephalon, we reconstructed a superior longitudinal fasciculus complex (SLFc) encompassing the three branches of the SLF usually defined in macaques, as a complex of dorsal longitudinal fibers connecting the frontal lobe with parietal and posterior temporal cortices. We observed that in all three species the SLFc terminates posteriorly in the posterior parietal cortex, but in rhesus macaques and squirrel monkeys the SLFc projects further rostrally than in ring-tailed lemurs (Fig. 9*A,B*). This can also be seen when comparing the location of the prefrontal cortex and the SLFc projection on the cortical surface (Fig. 10). Indeed, it would appear that the SLFc in ring-tailed lemurs shows weaker frontal connectivity, possibly due to the reduced size of their PF.

The optic radiation (OR) was reconstructed as a tract that connects the lateral geniculate nucleus of the thalamus and the primary visual cortex. Its anatomy in the different species confirms the previous observation made on cortical labeling of V1, as this tract’s posterior projections highlight the extent of V1. The relative enlargement of V1 in the calcarine fissure in the squirrel monkey is evident also from the OR projections. The lateral position of macaque V1 is also accompanied by weak lateral projections of OR in this species (Figs 9*A,B* and 10).

To confirm the differences observed in SLFc and OR, namely that SLFc connectivity is reduced in ring-tailed lemurs frontal cortex, and OR is disproportionately expanded in squirrel monkeys, we performed a ratio analysis with tracts that are similar between the different species and running in similar areas (dorsal and temporo-occipital). The CB runs in both dorsal and temporal areas and is a tract usually considered to be conserved across species (Bubb et al. 2018). Therefore, we performed the ratio of our two tracts of interest with the CB section that runs in a similar territory. These analyses confirmed our observation that the SLFc is proportionately smaller in ring-tailed lemurs, and it also revealed that the macaque SLFc seems proportionately smaller than that of the squirrel monkey SLFc (Fig. 9*C*). The OR was demonstrated to be proportionately larger in squirrel monkeys, while a very similar ratio with CBt was obtained for ring-tailed lemurs and rhesus macaques.

## Discussion

In the current study, we investigated cortical morphology and white matter architecture in three species of primates, representing three distinct lineages within the order, ring-tailed lemurs (which belong to the suborder Strepsirrhini, family Lemuroidae, also referred to as a prosimian primate), black-capped squirrel monkeys (which belong to the suborder Haplorrhini, parvorder Platyrrhini, family Cebidae, also referred to as New World monkey, simian primate or anthropoid primate), and rhesus macaques (which belong to the suborder Haplorrhini, parvorder Catarrhini, family Cercopithecidae, also referred to as Old World monkey, simian primate or anthropoid primate). To our knowledge, we present the first reconstructions of white matter tracts in the ring-tailed lemur, as well as expanding our knowledge of these tracts in the squirrel monkey. The white matter tracts show a generalized similarity across the primate species studied here and those studied previously. Our results identified an elaboration of prefrontal and parietal connectivity in squirrel monkeys and rhesus macaques (simians) compared to ring-tailed lemurs (prosimians), among a range of variations that might be considered to represent a simian *versus* prosimian divergence in cortical organization. We also observed a very specific occipito-parietal anatomy in the squirrel monkey that distinguishes the squirrel monkey from the other species studied.

Despite these variations, the white matter tracts show a generalized similarity across the primate species studied here and other primates. Mammals have been shown to share fundamental principles of brain connectivity such as wiring costs and speed of conduction, and more recently, a conserved relationship in which fewer interhemispheric connections are associated with better intrahemispheric connectivity and *vice versa*, maintaining the overall efficiency of communication across a large range of species (Assaf et al. 2020). The addition of new cortical association fields in the different lineages of primates contributes to our knowledge of the evolutionary specializations of this order (Krubitzer 2009). However, the overall principles of white matter organization appear conserved across the primate order (Rilling et al. 2008; Barrett et al. 2020; Eichert et al. 2020; Roumazeilles et al. 2020) despite differences in brain size and sulcal pattern, representing what might be an order-specific organization of the white matter (Manger 2005). The seven white matter tracts studied here are consistent with this pattern, showing a conserved organization in terms of topological relationship and connectivity of comparable brain regions, across three primate species that represent three distinct lineages within the order. Despite this overall similarity, interspecific variations were observed, particularly in the connectivity of association areas.

The identified differences between prosimian and simian primates were mainly associated with the frontal and parietal cortical areas. Prefrontal cortex has been previously reported to be proportionately larger in simians than prosimians, with several granular prefrontal cortical areas reported to be present only in simians (Preuss and Goldman-Rakic 1991). We have illustrated these findings here on the surface brains we created for the prosimian and the two simians. The comparative neuroimaging analysis undertaken in the current study provided further supportive evidence for these suggestions in terms of white matter organization. Particularly, although we could reconstruct the SLF in all species studied, there were less extensive frontal projections in the prosimian, possibly reflecting a weaker frontal connectivity, which is consistent with the limited size of the prefrontal cortex reported in prosimians. Similarly, the frontal projections of the IFOF appear less robust in the prosimian. In contrast, the UNC, which projects to the granular prefrontal areas that are present in all three species studied, appeared to reach the frontal pole more in the prosimian. This illustrates the reduced size of frontal granular areas observed in prosimians, confined only to the frontal extremity while extending more posteriorly in simians. We also noticed differences in the anatomy of the white matter associated with the parietal cortex when comparing between prosimians and simians. The ILF showed more limited posterior projections in the prosimian as it did not reach the parietal cortex, whereas it did in the two simians studied. In addition, the CB showed projections to the posterior parietal region in the two simians but not in the prosimian. These frontal and parietal variations between prosimians and simians support previous findings showing that the connectivity between frontal, parietal, and temporal areas is more complex in simian primates compared to prosimian primates, giving rise to what has been termed the “anthropoid elaboration” (Krubitzer 2009).

The additional granular prefrontal cortical areas in simian primates have been postulated to be associated with certain ecological and social factors and their abilities to forage in a more complex niche than prosimian primates. It has been further argued that these prefrontal networks have increased access to posterior parietal cortex information related to relational metrics, using them for more general decision making processes (Genovesio et al. 2014). The findings of the reduced parietal ILF projections in prosimians raise similar questions. Based on tracer work in the macaque monkey, Schmahmann and Pandya (2006) identified the ILF as a long association fiber tract of the ventral visual pathway in the occipitotemporal cortices. Importantly, they also highlighted its connectivity with parietal cortex, suggesting the importance of integrating the attentional functions of these areas into the visual processing of the ventral stream. Although the current state of knowledge and methods does not allow us to link white matter tracts and functions with certainty, it is interesting to discuss this observation in light of the known functions of areas associated with the ILF. Kaas (2017) highlights an expansion of the ventral visual stream in simians, presumably to enhance the ability to recognize individual faces and objects. The ILF has been specifically associated with providing visual input to this system for facial recognition (Herbet et al. 2018) and it is therefore striking that a species such as the lemur, usually living in smaller groups and relying on audition and olfaction in addition to vision to recognize individuals, would possess a reduced ILF (Kulahci et al. 2014). Furthermore, we have previously showed that the ILF is even more complex in great apes, where it is divisible into two subcomponents, the most ventral of which is homologous to macaque ILF and reaches face-responsive areas in all of the anthropoids species studied (Roumazeilles et al. 2020).

Our study also highlights specific features of the occipital region for the squirrel monkey. The size of the primary visual cortex, and more generally occipital cortex, is particularly large in the squirrel monkey compared to the two other species studied. This is associated with the posterior projections of the OR projecting more broadly than in the two other species. We also note other subtler differences in tract projections to occipital regions in squirrel monkeys compared to the two other species, including broader MdLF projections and more restricted IFOF projections. In this context, it is also interesting to note that the ILF appears more dominant in the posterior parietal area in squirrel monkeys than in macaques. These anatomical details in the relative size of different cortical regions and the projections of the associated tracts suggest a potential specialization of the occipital cortex in squirrel monkeys that has not been observed in other primates studied to date. Although all three species examined in this study are diurnal, squirrel monkeys have a lifestyle that is more arboreal-dependent than the two other species. Squirrel monkeys rarely come to the ground, and live and travel through small branches of trees, implying a challenge for neuronal information processing in both visual and locomotor systems. Interestingly, it has been previously noted that arboreal rodents devote more cortical territory to visual processing when compared to their terrestrial counterparts (Campi and Krubitzer 2010).

## Limitations and Future Studies

It should be noted that changes in proportion of white matter across the brains of different species may be due to allometric scaling (Ventura-Antunes et al. 2013). Inferring departures from allometric scaling rules generally requires analyses that take phylogenetic relationships into account and that include many more species than examined in the present study (Barton and Venditti 2013). However, the differences reported here are not differences in the relative quantity of cortical white matter, but rather qualitative variances between the cortical territories of a certain white matter tract. We have previously shown that qualitative variations, such as invasion of new cortical territories by a particular white matter tract, can be distinguished from cortical reorganization due solely to the expansion of the brain (Eichert et al. 2018, 2020). Our results are in line with such reorganizations of the intracortical white matter, showing that there are changes in the relative proportion of tracts in the same cortical areas, such as in the case of the SLFc and the dorsal cingulum, and that these tracts may project to novel parts of the cortex, such as in the case of the parietal connections of ILF in simians.

Tractography is a suitable tool to compare connectivity in different brains, as it allows for standardized protocols that are readily comparable across species; however, tractography is not without its caveats. First, probabilistic tractography applied to diffusion MRI data has been criticized recently for generating false positives and false negatives if not used with proper anatomical constraints (Maier-Hein et al. 2017). Here, we used previously validated protocols in macaques that have been shown to reconstruct tracts accurately (Warrington et al. 2020). Importantly, we were able to use three subjects per species and thus average the individual tractograms and threshold the result, which reduces the probability for false positives and negatives to be conserved in the final tract. Second, finding anatomical landmarks corresponding across species can sometimes be difficult, due to their very different overall anatomy. Taking several points of reference, both cortically and subcortically, which are known to have only limited variance across species, allows for a better approximation of the landmarks. Our tractography results also showed remarkable consistency with inferences made from the size and location of known cytoarchitectonic areas. Such convergence of results across modalities increases our confidence in the validity of our findings (Mars et al. 2021). It is also important to note that some of the tracts we delineated are very similar across species, such as the CB, MdLF, and IFOF. These tracts run in similar regions of the white matter to those for which we identified variations across species and were obtained based on similar landmarks. This reinforces confidence in our results. In addition, the reduced SLFc connectivity in lemur prefrontal cortex cannot be explained by reduced quality in the diffusion data in the frontal part of these brains, since the UNC seems to readily invade this space. It is also interesting to note that similar reduced SLFc connectivity has been observed in another prosimian, the galago (Bryant et al. 2021). Last, we have conducted control analyses when finding differences between species for the ILF, SLFc, and OR. Tracking from different areas and investigating appropriate ratios have confirmed our results.

Large-scale comparative neuroscience is often difficult, due to the labor and costs involved. MRI allows one to partially address these issues, but at present high-quality data are only available from a limited number of species. These data are usually acquired *postmortem* which could lead to tissue shrinkage, however this has a limited impact on the final data revealing higher levels of details in *postmortem* compared to in vivo imaging (Barrett et al. 2020). Although this study improves on previous methods by studying three individuals for each species, this number is still limited and does not allow for further interindividual and intersexes investigation, as for instance we could only obtain male ring-tailed lemurs. Fortunately, the situation is rapidly improving facilitated by the increasing collaborative nature of data sharing in nonhuman primate research and the inclusion of more species (Assaf et al. 2020; Milham et al. 2020; Bryant et al. 2021), leading to the requirement for standardized acquisition protocols, templates, and analysis strategies across species. The availability of multiple individuals with high-quality data from each species allowed us to create species-specific templates, in effect creating a “standard space” for each. All tractography protocols were defined in these standard spaces in a manner compatible with the recently released Xtract tools. All these resources are made available online to facilitate the easy exchange of information. Combined with other efforts using the same tools, this initiates more formalized phylogenetic comparisons of brain organization across the primate order.

## Conclusion

In summary, our study provides evidence in the form of white matter anatomy that supports the concept of the elaboration of prefrontal and posterior parietal systems in simians compared to prosimians. This study provides a baseline from which further studies, using similar standardized methods, can accurately compare brains across different species. This is of interest to allow us to understand the various evolutionary trajectories that influenced the structure of the brain within the different primate lineages and species.

## Notes

We thank Prof. Nicola R. Sibson for her support of the MRI scans. This research was funded in whole, or in part, by the Wellcome Trust [203139/Z/16/Z]. For the purpose of open access, the author has applied a CC BY public copyright license to any Author Accepted Manuscript version arising from this submission. *Conflict of Interest*: None declared.
